# Notes and News

**Published:** 1898-05-07

**Authors:** 


### Notes and News.

(Collected and Communicated.)

### HOSPITAL MEETINGS.

Free Home for the Dying.—Annual General Meeting, Monday, May 16th, at 4 p.m., at the Church House, Westminster.

Hospital for Women.—Annual Meeting, Thursday, May 19th, 3 p.m., at the Hospital.

North-Eastern Hospital for Children.—Annual Meeting, Monday, May 16th, 4 p.m., at the Hospital.

The sum of £300 has been granted from the estate of the late Mr. Richard Gibbs for the new science laboratories of the London School of Medicine for Women.

The Duke of Fife announced at the festival dinner of the Great Ormond Street Hospital that this institution has relieved nearly three-quarters of a million little patients during its career. Donations at the dinner amounted to £6,578, of which £4,500 was towards the purchase of the new site.

At the last meeting of the London School Board it was decided to appoint two medical practitioners to take charge of the work in connection with the examination of defective children and children alleged to be defective. The appointments are to be made provisionally for one year. Each practitioner is to give an equivalent of at least half time; one of them is to be a man and the other a woman; and the salary in each case is not to exceed £250 per annum.

According to the Rome correspondent of the *Lancet*, a circular has been addressed by the Italian Under-Secretary of State for the Home Department, in which "may be recognised the death-knell of the Santini Bill, which had for its object to exclude from practice in Italy all English-speaking medical men not provided with an Italian diploma." What this circular does is to urge the prefects throughout the kingdom to "exert a prompt and effective surveillance to the end that, except in special cases for which these physicians and surgeons are expressly summoned from abroad, the said practitioners, if they have not obtained the diploma of Italy, should restrict themselves to vouchsafing professional assistance to foreigners only." By implication, no doubt, this may be taken as a permission to practise among foreigners. What is clear, however, is that it is only a permission, which can be withdrawn at any time, and is by no means the establishment of a right.

The new issue of the "British Pharmacopœia" is now on sale, and the price has been fixed at 10s. 6d.

The miners at Crook have agreed to subscribe one halfpenny each every week for the maintenance of the proposed accident hospital, towards which Messrs. Pease have promised £500.

Sir Joseph Fayrer, Bart., K.C.S.I., M.D., LL.D., F.R.S., has accepted the presidency of the seventeenth Congress of the Sanitary Institute to be held in Birmingham, commencing September 27th.

The Lord Mayor, in appealing for funds at the festival dinner of the Metropolitan Hospital, called attention to the debts and mortgages which oppress the institution, and, "as a man of Kent," recorded his gratitude to the committee for having placed their convalescent home at Cranbrook at the service of the typhoid fever victims at Maidstone.

Various gipsy encampments are at the present time causing much disturbance in the minds of sanitary authorities. A colony of Servian gipsies, including ten bears, encamped at Edmonton, have been breaking every law of health and decency, and it is quite clear that some special legislation will shortly have to be enacted to deal summarily with such sanitary offenders. The writer recently saw a most gruesome collection of foreign and British vagrants entrenched and picnicing more or less permanently in close and dangerous proximity to the settling-tanks of a Kentish water-supply. There were all the elements in the situation of a typhoidal outbreak similar to that of Maidstone.

We sympathise and condole with Dr. Coley, who is so well known for his investigations in regard to the influence of the toxins of erysipelas upon the progress of sarcoma. It appears that a certain Vaccine Company of Chicago has not only used Dr. Coley's name without his consent in connection with a preparation of the toxins of erysipelas and prodigiosus which it has placed upon the market, but has spoken of this substance as a remedy for cancer, and as "especially adapted for use in the early stages of the disease." This is too bad. Not only do they make an unauthorised use of Dr. Coley's name, but they misrepresent the very position he has taken up, for he has never recommended these toxins except in inoperable cases, and practically only in cases of inoperable sarcoma.

The inhabitants of New York have lately awakened to the fact that their city is conspicuously below the standard of other civilised cities in the question of street cleanliness. Stringent measures have lately been taken to add to the hygiene of streets and pathways. Now, any citizen who throws paper, banana skins, or any other rubbish on to the roads is liable to arrest and fine. Two small boys were recently arrested and charged with the offence of scattering pieces of newspaper in their path. They were convicted as a warning to all the other young citizens, and as a reminder that each has a civic duty to perform in adding his mite to the healthiness and well-being of the place in which he lives. Arrest for so slight an offence seems somewhat of a stringent measure; but it will undoubtedly point the moral of sanitation.

